# From Limb Salvage to Prosthetic Rehabilitation After Severe Traumatic Hand Injury Complicated by Complex Regional Pain Syndrome: A Case Report

**DOI:** 10.3390/jcm15208000

**Published:** 2026-10-15

**Authors:** Ozana Katarina Tot, Anja Petričević, Dijana Hnatešen, Ana Cicvarić, Iva Dimitrijević, Vanja Matković

**Affiliations:** 1 Faculty of Medicine Osijek, Josip Juraj Strossmayer University of Osijek, 31000 Osijek, Croatia; 2 Department of Anesthesiology, Intensive Care Medicine and Pain Management, University Hospital Centre Osijek, 31000 Osijek, Croatia; 3 Palliative Care Department, Osijek-Baranja County Health Center, 31000 Osijek, Croatia; 4 Department of Health Studies, Lavoslav Ružička Polytechnic in Vukovar, 32000 Vukovar, Croatia

**Keywords:** complex regional pain syndrome, traumatic hand injury, amputation, multidisciplinary rehabilitation, prosthetic rehabilitation

## Abstract

**Background/Objectives**: Severe traumatic injuries of the upper extremity present a major therapeutic challenge because of extensive tissue damage, severe pain, loss of function, and substantial psychosocial consequences. Continuous multidisciplinary rehabilitation that adapts to the patient’s evolving clinical condition may optimize functional outcomes across the entire continuum of care. **Methods**: A 42-year-old man with no prior comorbidities sustained a severe work-related traumatic injury to his left hand. Despite reconstructive surgery and prolonged multidisciplinary treatment, he developed CRPS with persistent neuropathic pain, progressive functional deterioration, and psychological distress. Fourteen months after injury, the affected limb became completely nonfunctional, and elective transradial amputation was recommended after multidisciplinary evaluation. **Results**: Rehabilitation was delivered as a continuous, phase-adapted multidisciplinary program. Before amputation, treatment focused on pain control, reduction of edema and inflammation, preservation of joint mobility and muscle function, and management of CRPS using multimodal physical therapy combined with pharmacological treatment and psychological support. As amputation became inevitable, rehabilitation goals shifted toward pre-amputation preparation, promotion of adaptive neuroplasticity, and prevention of phantom limb pain. After transradial amputation, pain intensity markedly decreased, functional adaptation improved, and the patient successfully completed prosthetic rehabilitation. **Conclusions**: This case highlights the importance of a continuous, individualized multidisciplinary rehabilitation strategy extending from limb salvage through post-amputation recovery. Integrating multimodal physical therapy, neuroplasticity based interventions, psychological support, and prosthetic rehabilitation may improve pain control, facilitate functional recovery, and enhance long-term adaptation after severe traumatic upper extremity injury.

## 1. Introduction

### 1.1. Traumatic Upper Limb Amputation and the Role of Multidisciplinary Rehabilitation

Traumatic upper limb amputation represents a relatively small proportion of all amputations but leads to disproportionately greater functional impairment. This is due to the complex motor and sensory functions of the hand and upper limb, altered body image, reduced independence in activities of daily living, and a substantial psychosocial burden [1]. In addition to the immediate loss of limb function, patients may experience residual limb pain, phantom limb pain (PLP), anxiety, depression, reduced acceptance of the prosthesis, and difficulty returning to work, underscoring the need for comprehensive multidisciplinary rehabilitation from the earliest stages of treatment [2].

Rehabilitation after traumatic injury of the upper extremity should not be viewed as a process that starts only after amputation. Whenever limb salvage becomes uncertain, pre-amputation rehabilitation can help preserve joint mobility, maintain muscle strength, reduce pain and edema, plan for secondary complications, and prepare patients physically and mentally for surgery and subsequent prosthesis fitting [3]. Early rehabilitation is also a key factor in successful prosthesis adaptation, long-term prosthesis acceptance, and the restoration of functional independence [1]. After amputation, the goals of rehabilitation are to optimize wound healing, shape the residual limb, prevent contractures, facilitate prosthetic training, and maximize functional recovery [4]. Emerging evidence further suggests that early rehabilitation may attenuate maladaptive neuroplastic changes associated with chronic pain syndromes, particularly phantom limb pain [5].

### 1.2. Multimodal Physical Therapy and Neuroplasticity in Post-Amputation Rehabilitation

Physical therapy modalities, including transcutaneous electrical nerve stimulation (TENS), therapeutic ultrasound, low-level laser therapy (LLLT), and pulsed electromagnetic field therapy (PEMF), are commonly incorporated into multimodal rehabilitation programs to support pain relief, tissue healing, and functional recovery. Their complementary biological effects may target pain, inflammation, tissue repair, and restoration of function [6]. TENS provides analgesia through activation of large-diameter Aβ afferent fibers, inhibition of nociceptive transmission, descending pain modulation, and endogenous neurotransmitter release. A 2022 meta-analysis by Johnson et al. supported its use as a safe, nonpharmacological adjunct for pain management [7,8]. Its favorable safety profile and ease of application make it suitable for both preoperative and postoperative rehabilitation [9].

Therapeutic ultrasound produces thermal and nonthermal effects that may promote local circulation, fibroblast activity, collagen synthesis, angiogenesis, and tissue repair, while reducing muscle spasm and improving connective tissue extensibility. Its clinical benefits depend on the underlying pathology and treatment parameters [10,11]. Photobiomodulation therapy (PBM/LLLT) acts primarily through mitochondrial photoreception, modulating ATP production, reactive oxygen species, and cellular signaling pathways involved in analgesia, inflammation, wound healing, and tissue regeneration [12,13,14]. PEMF may influence ion transport, intracellular calcium signaling, nitric oxide synthesis, and cellular metabolism, potentially supporting microcirculation, reducing inflammation and pain, and promoting tissue healing [15,16]. When individualized and integrated into rehabilitation programs, these modalities may contribute to improved functional outcomes, although their effects depend on the clinical context and treatment protocols [17].

Functional recovery following injury also depends on adaptive neuroplastic changes involving interactions between peripheral sensory input and central nervous system processing, which influence motor control, body representation, and pain perception [18]. Rehabilitation should therefore address both peripheral tissue recovery and central sensorimotor adaptation, particularly in patients at risk of phantom limb pain [19]. After amputation, neuroplasticity enables the reorganization of cortical representations and the formation or strengthening of neural connections that support functional recovery [20,21]. However, the permanent disruption of afferent input may also induce maladaptive changes in sensorimotor networks, affecting cortical excitability, body representation, motor planning, and pain processing [22]. Cortical remapping, in which stimulation or movement of other body regions activates cortical areas that formerly represented the amputated limb, is a well-documented manifestation of this plasticity [23]. Although cortical reorganization may be adaptive, excessive or maladaptive changes have been associated with disturbed body representation, sensorimotor mismatch, and persistent neuropathic pain [24]. Accordingly, rehabilitation should aim to restore physical function while promoting adaptive cortical reorganization and minimizing maladaptive neuroplasticity [25].

### 1.3. Phantom Limb Pain, Mirror Therapy, and Rationale for the Present Case

Phantom limb pain (PLP) is one of the most common long-term complications after limb amputation, affecting approximately 60–85% of amputees [26]. PLP is defined as painful sensations perceived in the absent limb and should be distinguished from painless phantom limb sensations and residual limb pain localized to the stump [24]. The pathophysiology of PLP is multifactorial and includes peripheral nerve injury, ectopic discharges within neuromas, spinal cord sensitization, altered thalamocortical processing, maladaptive cortical reorganization, and immunologic and psychological mechanisms [27]. Growing evidence suggests that persistent nociceptive input before amputation, together with postoperative pain, enhances these neuroplastic changes and contributes to the development of chronic phantom limb pain [28].

Among nonpharmacologic interventions, mirror therapy (MT) has emerged as one of the most extensively investigated neurorehabilitation techniques for managing phantom limb pain [29]. Mirror therapy, originally introduced by Ramachandran and Rogers-Ramachandran, provides visual feedback by reflecting the movements of the intact limb, thereby creating the illusion that the amputated limb is present and moving normally [23,30]. This visual illusion is believed to reduce the mismatch between motor intention and absent sensory feedback, facilitating restoration of the body schema and normalization of sensorimotor cortical networks [25].

Functional neuroimaging studies further show that successful mirror therapy is accompanied by measurable changes in cortical activation patterns, supporting the hypothesis that visual feedback can partially reverse the maladaptive cortical reorganization associated with phantom limb pain [31]. More recently, mirror therapy has been considered part of a broader spectrum of neurorehabilitation interventions that includes motor imagery, graded motor imagery, sensory discrimination training, and virtual reality-based rehabilitation, all of which aim to restore coherent sensorimotor integration and normalize cortical representation [19,20].

Recent systematic reviews, randomized controlled trials, and expert recommendations consistently support including mirror therapy in comprehensive rehabilitation programs for patients with phantom limb pain [29]. Although treatment protocols differ in session duration and therapy frequency during the intervention period, regular mirror therapy is associated with reduced pain intensity, enhanced motor imagery, improved cortical reorganization, and better functional adaptation to limb loss [18,29]. Furthermore, combining mirror therapy with conventional physical rehabilitation, early prosthetic fitting, and individualized multidisciplinary treatment may provide synergistic benefits by simultaneously addressing peripheral tissue recovery and central neuroplastic mechanisms [17,25].

Taken together, current evidence supports a multimodal rehabilitation approach that integrates conventional physical therapy modalities with interventions specifically targeting cortical reorganization to optimize outcomes after traumatic upper limb amputation [26].

Despite significant advances in surgical techniques, prosthetic technologies, and rehabilitation strategies, the optimal integration and timing of different physical therapy modalities across the continuum of care after traumatic upper limb amputation remain underexplored [17]. Most published studies have evaluated individual rehabilitation interventions, such as mirror therapy, prosthetic training, or pharmacological treatment of phantom limb pain, whereas relatively few reports have described a comprehensive rehabilitation program that begins before amputation, continues throughout the postoperative period, and integrates conventional physical therapy modalities with interventions specifically targeting neuroplasticity [32]. Consequently, clinical evidence on the potential synergistic effects of combining peripheral tissue-directed therapies with central nervous system-directed rehabilitation in patients undergoing traumatic upper limb amputation remains limited [17,25].

This case illustrates a multidisciplinary rehabilitation strategy applied to a patient with a severe traumatic upper limb injury that ultimately required amputation after unsuccessful limb salvage [17,32]. During the pre-amputation period, rehabilitation focused on pain control, inflammation reduction, preservation of joint mobility, and optimization of soft tissue healing through a multimodal therapeutic program [6,15]. After amputation, rehabilitation priorities shifted toward preventing secondary complications of the stump, facilitating adaptive cortical reorganization, and reducing phantom limb pain with mirror therapy [18,33].

This multimodal rehabilitation strategy reflects the contemporary understanding that successful recovery from limb amputation depends on addressing both peripheral biological processes and central neuroplastic mechanisms [33]. Conventional physical therapy methods can optimize tissue healing, reduce nociceptive input, and create favorable conditions for functional recovery during the acute and subacute phases of rehabilitation [6]. In contrast, mirror therapy primarily targets maladaptive cortical reorganization by restoring congruence between motor intention and visual feedback, thereby facilitating normalization of body representation and sensorimotor cortical networks [18]. Together with individualized prosthetic rehabilitation, these complementary interventions can promote functional adaptation, improve prosthesis acceptance, reduce phantom limb pain, and enhance overall quality of life [3,34]. The aim of this case report is to describe the continuity of multidisciplinary rehabilitation from limb salvage through transradial amputation to prosthetic rehabilitation in a patient with CRPS following severe hand trauma.

## 2. Case Presentation

This case report describes the clinical course and multidisciplinary rehabilitation of a patient with a severe traumatic upper limb injury that ultimately required amputation. Rehabilitation was provided as a phase-adapted, stepwise program that included multimodal physical rehabilitation before amputation, mirror therapy during the postoperative period, and structured prosthetic training during functional recovery. The report highlights the value of a continuous, phase-adapted multidisciplinary rehabilitation strategy that bridges limb-salvage management and post-amputation rehabilitation while integrating tissue-directed interventions with neuroplasticity-based therapies to support functional recovery.

A 42-year-old man with no prior history of peripheral neuropathy, chronic pain disorders, or psychiatric illness sustained a severe injury to his left hand while working on a woodworking machine at work in September 2021. The injury to fingers II to V of the left hand resulted in extensive soft tissue damage, skin defects, and tendon injuries to the flexor digitorum profundus of the second and fourth fingers. On the day of the injury, the patient underwent emergency reconstructive surgery under general anesthesia, including a cross-finger flap, plastica defecti cutis sec. Thiersch, and immobilization. General anesthesia was selected for the initial emergency surgical procedure because the patient, who had sustained a traumatic workplace injury, was highly distressed by the circumstances and severity of the injury and did not consent to regional anesthesia. General anesthesia therefore enabled the necessary surgical treatment while respecting the patient’s decision regarding the anesthetic technique.

**Figure 1:**
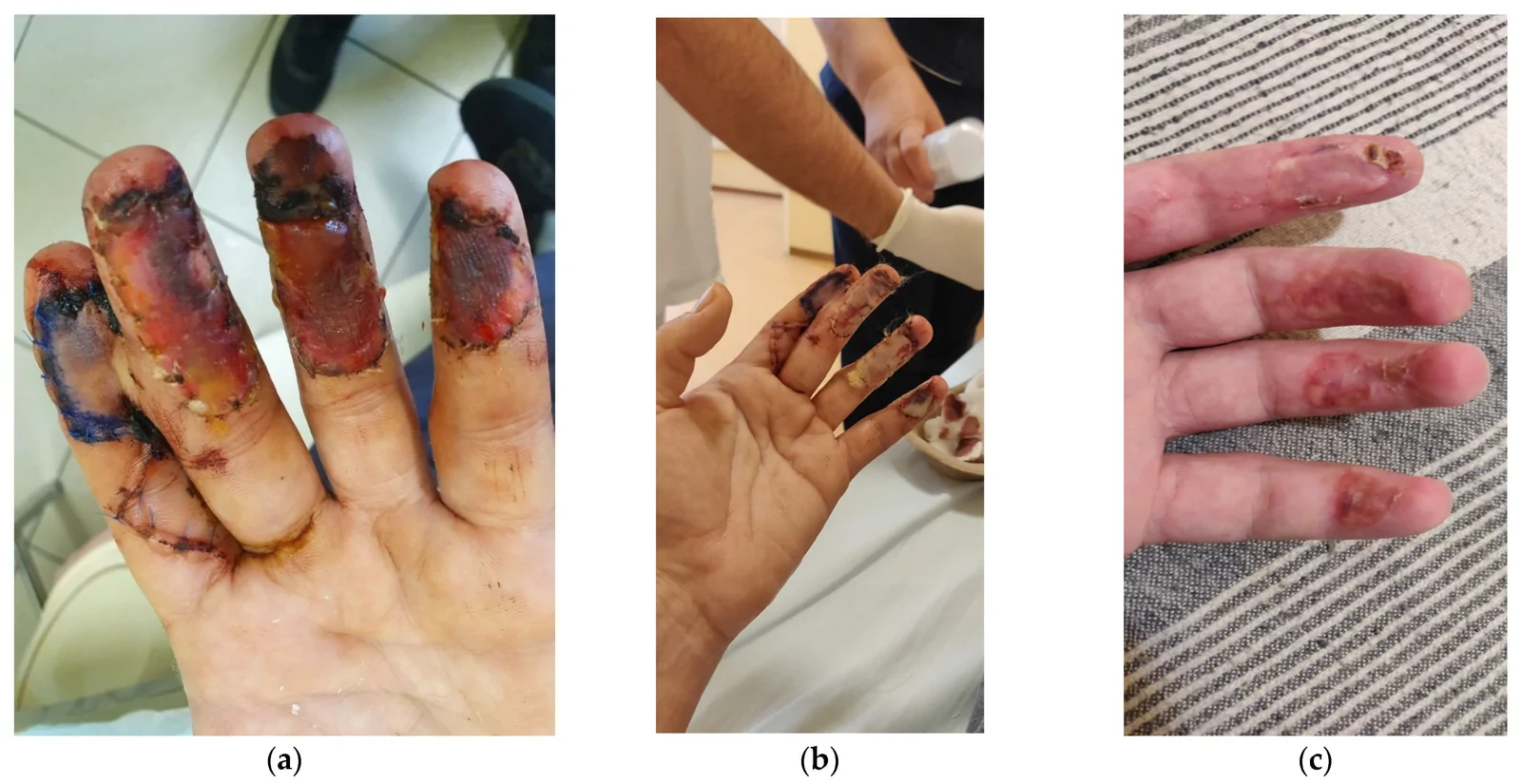
Clinical appearance of the left hand 4 weeks (**a**,**b**) and 2 months (**c**) after surgery, demonstrating the postoperative recovery process.

The early postoperative course was uneventful, and he was discharged on the fifth day with antibiotic therapy and routine wound care. Three weeks later, the flap was separated from the donor finger, and a secondary wound revision was performed under local anesthesia (Figure 1).

Despite satisfactory wound healing, functional recovery was slow. During follow-up examinations over the next months, the patient gradually developed severe neuropathic pain, hypersensitivity, persistent dysesthesia, paresthesia, burning sensations, cold intolerance, muscle cramps, progressive finger contractures, significant functional impairment, and hyperpigmentation of the affected hand. The pain was present both during activity and at rest, and its intensity progressively increased. During the postoperative rehabilitation period, the patient was encouraged to gradually resume functional activities as wound healing progressed and the condition of the repaired tissues allowed. However, despite analgesic treatment, the patient continued to experience severe pain accompanied by considerable anxiety and distress. This significantly limited the patient’s willingness to participate in therapeutic interventions and led to repeated refusal of manipulation of the left hand, complicating the planned progression of rehabilitation and reintegration into activities of daily living.

Based on the clinical findings, a diagnosis of complex regional pain syndrome (CRPS 1) was established according to the Budapest clinical diagnostic criteria [35]. The patient reported ongoing pain disproportionate to the initial injury and met the required symptom and sign categories, including sensory abnormalities (mechanical allodynia and hyperesthesia), vasomotor changes (temperature sensitivity), sudomotor/trophic changes (persistent edema and skin discoloration), and motor dysfunction (reduced range of motion, weakness, and progressive finger contractures). No alternative diagnosis better accounted for the clinical presentation.

**Figure 2:**
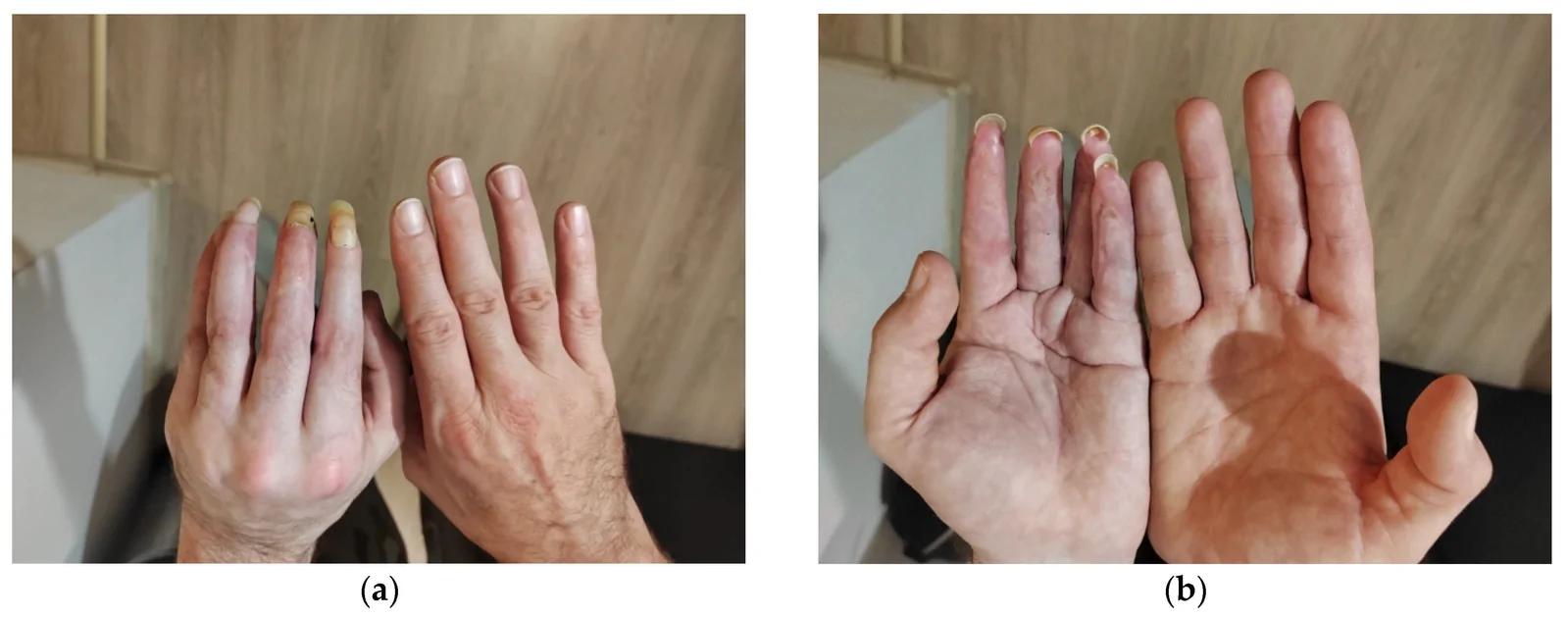
Clinical appearance of the left hand 6 months after surgery, demonstrating the functional and morphological outcome of rehabilitation (**a**,**b**).

Six months after the injury, the initial pain assessment showed continuous neuropathic pain rated on a numerical rating scale (NRS) between 7 and 10 (Figure 2).

At the plastic surgeon’s initiative, the patient was referred to the pain clinic in March 2022. Physical examination at that time documented pain rated 9 on the NRS, confirmed pronounced mechanical allodynia affecting the palm and fingers of the left hand, and led to the immediate initiation of multimodal physical treatment, including transcutaneous electrical nerve stimulation (TENS), magnetotherapy, low-level laser therapy (LLLT), ultrasound therapy, acupuncture, and pharmacological treatment combining pregabalin, gabapentin, nonsteroidal anti-inflammatory drugs, tramadol, duloxetine, and later tapentadol, depending on the progression of symptoms and pain (Table 1).

**Figure 3:**
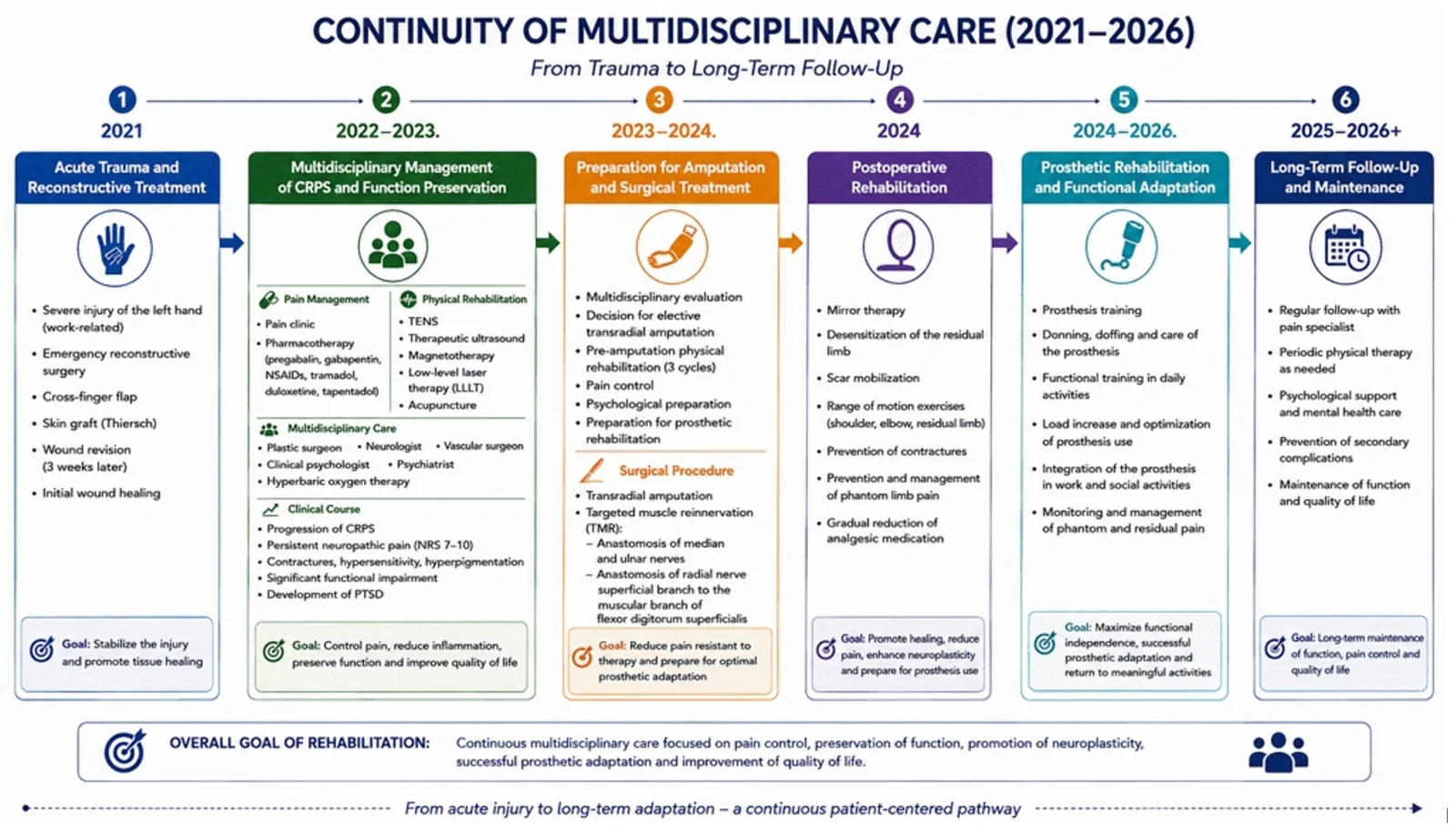
Timeline of the Patient’s Multidisciplinary Treatment and Rehabilitation Pathway from Injury to Long-term Follow-up.

From March 2022 to March 2023, four cycles of multimodal physical treatment were administered, producing temporary improvement by reducing pain to 7 on the NRS, but the pain consistently recurred and functional deterioration continued (Figure 3).

**Figure 4:**
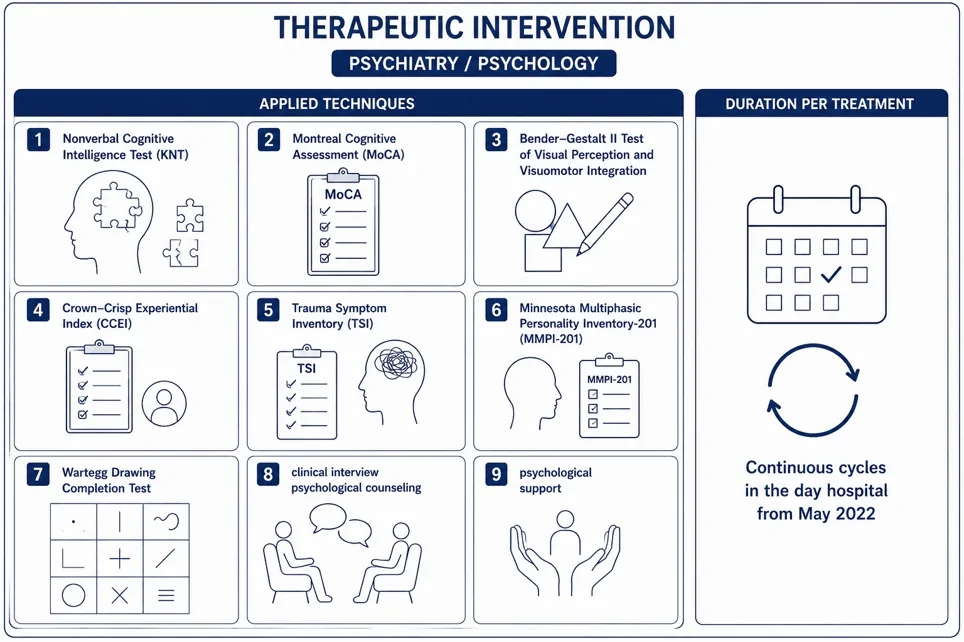
Psychiatry/Psychology intervention protocol.

Psychiatric evaluation revealed the development of an adjustment disorder followed by posttraumatic stress disorder (PTSD) associated with the work-related injury. Symptoms included intrusive memories, sleep disturbance, anxiety, depressed mood, irritability, emotional withdrawal, reduced frustration tolerance, and significant deterioration in quality of life. From May 2022 to February 2024, he underwent cyclical psychiatric treatment in a day hospital setting, along with structured psychotherapy and antidepressant and anxiolytic medication (Figure 4).

Psychiatric and psychological management consisted of continuous day-hospital treatment cycles starting in May 2022. The protocol included standardized neuropsychological and psychodiagnostic assessments using the Nonverbal Cognitive Intelligence Test (KNT), Montreal Cognitive Assessment (MoCA), Bender–Gestalt II Test of Visual Perception and Visuomotor Integration, Crown–Crisp Experiential Index (CCEI), Trauma Symptom Inventory (TSI), Minnesota Multiphasic Personality Inventory-201 (MMPI-201), and the Wartegg Drawing Completion Test, followed by clinical interviews, psychological counseling, and ongoing psychological support throughout the rehabilitation program.

Follow-up examinations by a plastic surgeon documented progressive extension contracture of the proximal and distal interphalangeal joints, persistent weakness of active finger movements, sensory hypersensitivity, and loss of hand function. Hyperbaric oxygen therapy was recommended for 10 sessions, which did not significantly improve symptoms. Clinical evaluation by a vascular surgeon noted preserved radial and ulnar arterial circulation with no indication for vascular reconstruction, despite reduced capillary refill in the affected fingers. Neurological evaluation revealed severe neuropathic pain associated with hypersensitivity and functional impairment; however, somatosensory evoked potentials of both median nerves remained within normal limits. During follow-up examinations by a plastic surgeon, an algologist, and a neurologist, the patient continued to report severe burning pain, temperature hypersensitivity, numbness, and progressive inability to use his left hand. By October 2023, despite long-term multidisciplinary treatment that included reconstructive surgery, physical rehabilitation, pain management, neurology, vascular surgery, clinical psychology, and psychiatry, the patient continued to experience unbearable pain with NRS 9 intensity, associated with complete functional loss of the affected limb and progression of PTSD. Evaluation at the Center for Plastic Surgery and Hand Reconstruction concluded that further reconstructive procedures were not feasible. After multidisciplinary discussion and detailed counseling, the diagnosis was made: Sudeck antebrachii lat. sin. syndrome. From October 2023 to January 2024, three additional cycles of multimodal physical therapy, each lasting 15 days, were conducted to reduce the risk of developing phantom pain and to facilitate subsequent prosthetic rehabilitation. After available pharmacological and physical treatment options had been exhausted, cervical spinal cord stimulation was offered as a potential treatment; however, the patient declined this intervention and continued to express a strong preference for amputation. This decision followed several discussions with the treating plastic surgeon and took into account the patient’s persistent pain, functional impairment, emotional distress, previous treatment experiences, and clearly stated preferences.

Elective amputation was recommended due to therapy-resistant pain and a completely nonfunctional hand.

In February 2024, to reduce the risk of symptomatic neuroma and recurrent neuropathic pain, the patient underwent left transradial amputation with intraoperative targeted nerve reconstruction consisting of amputatio antebrachii sin. with anastomosis of the n. medianus and n. ulnaris antebrachii of the left hand, and anastomosis of the muscularis muscle of the flexor digitorum superf. cum n. radialis r. superficialis.

**Figure 5:**
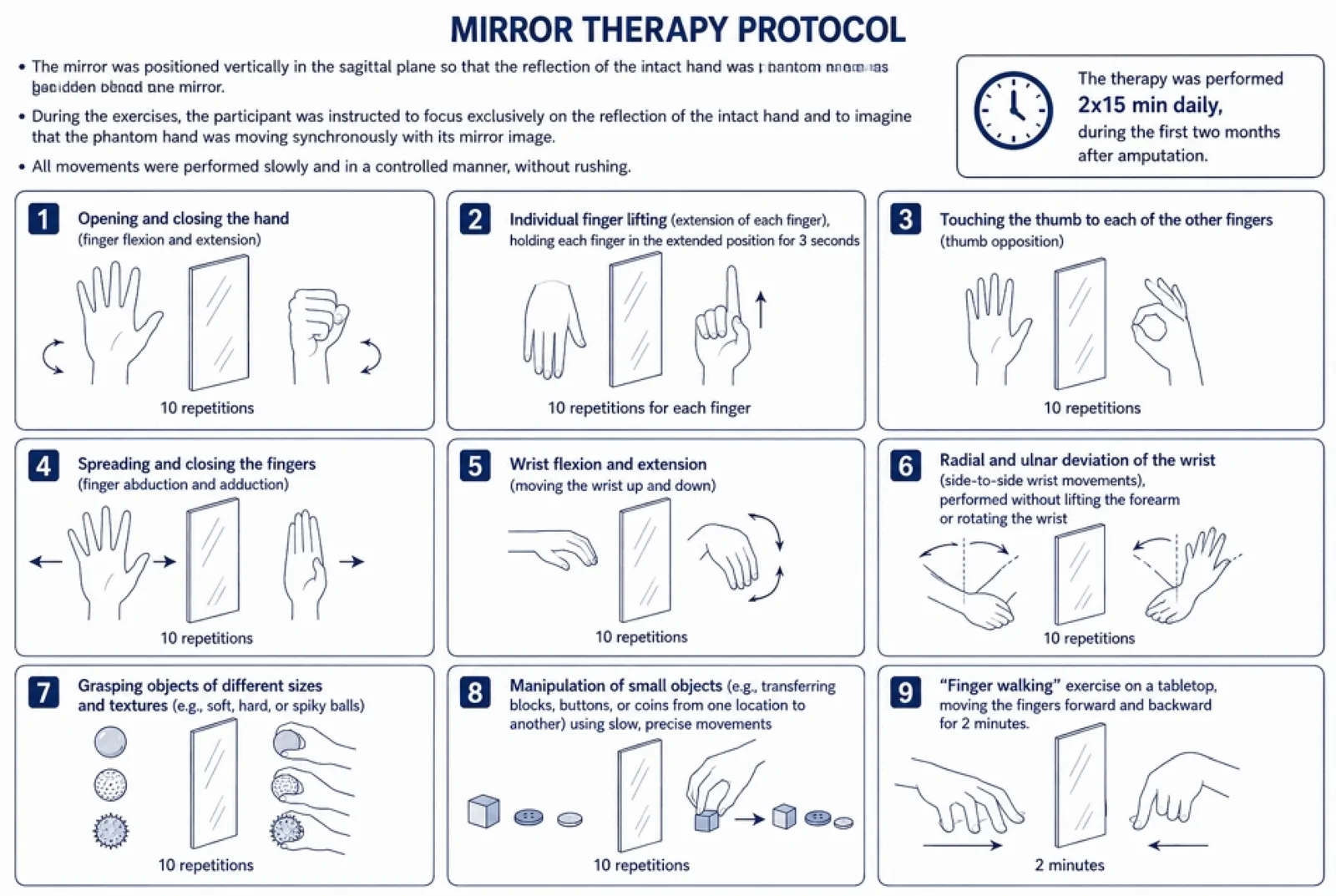
Mirror therapy protocol used during the first two months following transradial amputation.

At the first postoperative follow-up, 3 weeks after transradial amputation of the left forearm, the patient reported a significant decrease in pain intensity (numerical rating scale NRS 3) compared with the preoperative state. Residual symptoms consisted of occasional stabbing pain and intermittent paresthesia within the patella. Pharmacological treatment was gradually tapered over the next 6 months, with reduction in the doses of pregabalin, duloxetine, and opioid analgesics until complete discontinuation of pregabalin and duloxetine 2 months after amputation. During the first 2 months after amputation, the patient followed a structured home rehabilitation program focused on mirror therapy, stump desensitization, shoulder and elbow range-of-motion exercises, scar mobilization, and gradual functional adaptation guided by a physiotherapist (Figure 5).

Therapy was administered twice daily for 15 min. The mirror was positioned in the sagittal plane so that only the reflection of the intact hand was visible. The patient was instructed to focus on the mirror image while imagining synchronous movement of the phantom limb. Exercises progressed from simple hand and wrist movements to object manipulation and fine motor tasks. All movements were performed slowly and in a controlled manner.

**Figure 6:**
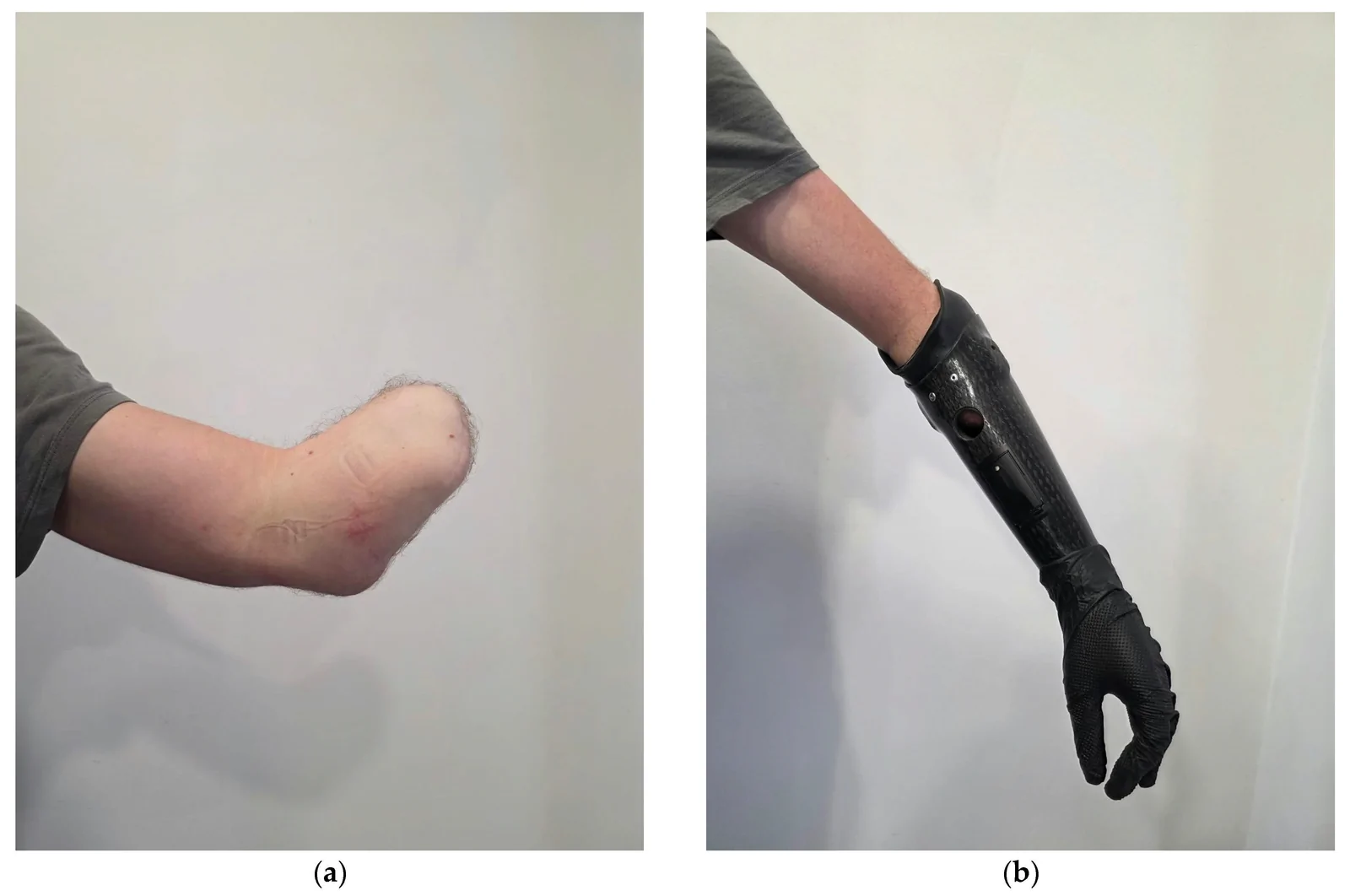
Functional prosthetic rehabilitation after transradial amputation of the left forearm. (**a**) Functional use of a forearm prosthesis with a myoelectric hand. (**b**) Residual limb of the left forearm prepared for prosthetic fitting.

Mirror therapy was performed daily according to the rehabilitation team’s instructions and continued until the start of formal prosthetic rehabilitation. Prosthetic rehabilitation was initiated in June 2024. In August 2024, the patient completed a one-week inpatient rehabilitation program at a specialized prosthetic rehabilitation institute, where he received structured training and education in the use and functional application of a forearm prosthesis with a myoelectric hand (Figure 6).

The patient continued to be followed by an algologist through follow-up examinations every 3 months, reporting stable residual stump pain related to the weight of the prosthesis, with occasional phantom limb pain (NRS 3–5) compared with the pain before amputation (NRS 7–9). Intermittent multimodal physical therapy was continued every 6 months for symptom control and management of pain in the phantom hand of the left upper limb. The patient also continues to participate in a day hospital program with monitoring by a psychologist and psychiatrist (Table 2).

## 3. Discussion

This case report describes a complex, long-term, multidisciplinary rehabilitation process in a patient with a severe traumatic upper extremity injury that, despite treatment and rehabilitation, ultimately required transradial amputation. The uniqueness of this case lies in the continuous rehabilitation approach that encompassed all phases of care from acute injury management, through the period before amputation and early postoperative recovery, to prosthetic rehabilitation and return to functional activities. In the pre-amputation period, rehabilitation goals focused on preserving limb function, reducing pain and inflammatory response, controlling edema, maintaining joint mobility, preserving muscle activity, and optimizing soft tissue healing. To achieve these goals, a multimodal rehabilitation program was implemented, including transcutaneous electrical nerve stimulation (TENS), therapeutic ultrasound, magnetotherapy, low-intensity laser therapy, and acupuncture, combined with continuous psychological and psychiatric support through a day hospital program [36]. The use of different physical modalities in this phase was intended to modulate pain, improve local biological conditions for healing, and preserve the limb’s functional potential [32].

Deciding to amputate is among the most challenging aspects of treating patients with complex traumatic limb injuries and requires an individualized assessment by a multidisciplinary team [26]. In this case, the decision to perform a transradial amputation was made after determining that this approach could provide better overall function, lessen the long-term physical and psychological burden associated with the injured limb, and increase the likelihood of returning to independent living and work [37].

After amputation, rehabilitation priorities shifted toward preserving residual limb function, preventing secondary complications, promoting adaptive neuroplasticity, managing phantom limb pain, and preparing for prosthetic use [38]. Mirror therapy was incorporated into the rehabilitation program to facilitate sensorimotor adaptation and address pain mechanisms [39]. It aimed to stimulate sensorimotor reorganization in the central nervous system by reestablishing the connection between motor intention and visual sensory feedback [21]. Today, mirror therapy is one of the most frequently used non-pharmacological methods for treating phantom limb pain and for sensorimotor rehabilitation after amputation. Proposed mechanisms of action include modulation of maladaptive cortical reorganization, enhancement of sensorimotor integration, and reduction of abnormal neural activity patterns associated with phantom limb pain [30]. In this case, mirror therapy was introduced after the surgical wound had healed and was performed during the period before prosthetic rehabilitation, which is consistent with the concept of early implementation of interventions that target neuroplasticity after amputation [18].

Successful prosthetic rehabilitation is not just a matter of fitting a prosthesis, but a complex process that includes conditioning the residual limb, developing muscle control, learning new motor strategies, achieving functional performance in activities of daily living, and psychosocial adaptation to changed physical abilities. In this case, a structured prosthetic rehabilitation program was implemented with ongoing pain management, physical therapy, and psychological support, allowing a gradual increase in the patient’s functional independence. The relatively favorable functional outcome in this case is likely the result of the timely initiation and continuity of a multidisciplinary rehabilitation approach that encompassed different phases of recovery. The combination of peripheral tissue-focused interventions, neuroplasticity-based methods, and individualized prosthetic rehabilitation may contribute to better functional adaptation, greater prosthesis acceptance, reduced phantom and nociceptive pain, and improved quality of life after traumatic upper-extremity amputation.

In the present case, twenty months after amputation and completion of prosthetic rehabilitation, the patient returned to a work environment in a position adapted to his functional capabilities. However, despite satisfactory physical recovery, the psychological consequences of the traumatic event, including emotional reactions to the moment of limb loss and challenges associated with the social stigmatization of disability, remain a significant part of the recovery process. Therefore, long-term rehabilitation after amputation should not be limited to physical function alone, but should include ongoing psychological support, monitoring of anxiety symptoms, and adjustment to changing life roles [40]. Combining psychological interventions, such as structured therapeutic interviews and sensory feedback methods, with physical therapy and appropriate pharmacological support may offer additional benefits for long-term control of psychological symptoms and preservation of function.

## 4. Clinical Implications for Hand Therapy

This case underscores the importance of maintaining active involvement of hand therapists across the entire continuum of care following severe traumatic upper extremity injury, including situations in which limb salvage ultimately proves unsuccessful. Rehabilitation should not be viewed as separate pre- and post-amputation interventions but as a continuous, individualized process that evolves with the patient’s changing clinical needs. During limb salvage, hand therapy should prioritize pain management, edema control, preservation of joint mobility, maintenance of muscle function, sensory reeducation, and patient education. When amputation becomes inevitable, rehabilitation goals should gradually shift toward pre-amputation preparation, psychological support, prevention of secondary complications, promotion of adaptive neuroplasticity, and planning for prosthetic rehabilitation. After amputation, mirror therapy, residual limb desensitization, structured prosthetic training, and continued multidisciplinary follow-up may facilitate functional adaptation, reduce phantom limb pain, improve prosthesis acceptance, and support return to meaningful daily activities and employment.

## 5. Limitations

This case report has several limitations. Because multiple surgical, pharmacological, rehabilitation, and psychological interventions were used during treatment, it is not possible to precisely determine the specific contribution of each method to the overall functional outcome. In addition, the conclusions are limited by the fact that this is a single-patient report and cannot be directly generalized to a broader population. Furthermore, validated upper-extremity functional outcome measures (e.g., QuickDASH or PRWE) were not prospectively collected, limiting the objective assessment of functional improvement over time.

## 6. Patient’s Perspective

“After my arm was amputated, my life changed completely. Although deciding to undergo the amputation was very difficult for me, over time I realized it was the only way to relieve the unbearable pain and begin living a more normal life again. Today, the pain is much less intense than before the amputation, but it still occasionally appears as phantom or stump pain of varying intensity and duration. I have learned to live with these symptoms, although they remain part of my everyday life.

I have returned to a job that is adapted to my functional abilities and disability. This means a lot to me because it allows me to be active again and feel useful. However, daily work also brings new challenges. I feel that some of my colleagues have difficulty understanding the limitations I live with and sometimes expect me to perform the same tasks at the same pace as before the injury or as other employees. Such situations can cause frustration and remind me that the adaptation process is a challenge not only for me but also for the people in my work environment.

The psychological consequences of the injury and amputation are still present. That is why I regularly participate in a day hospital program where, with the help of a clinical psychologist, I undergo behavioral therapy and other forms of psychological support that help me cope with the trauma, anxiety, and changes the amputation has brought into my life.

I am aware that no one can predict with certainty how my symptoms will develop in the future. The pain still occasionally occurs with varying intensity, but I have learned to recognize its patterns and cope with them. I believe that ongoing physical therapy, regular checkups, and continuous psychological support will help me preserve my physical and mental health for as long as possible, maintain my functional independence and quality of life, and continue to live as actively as possible despite my permanent disability.”

## 7. Conclusions

This case report underscores the importance of a continuous, individualized, multidisciplinary rehabilitation approach after severe traumatic upper limb injury. Integrating procedures that promote peripheral tissue healing, neuroplasticity-based interventions, and structured prosthetic rehabilitation may provide a valuable framework for achieving optimal functional recovery and improving quality of life for patients after amputation. This case supports viewing rehabilitation as a continuous therapeutic process rather than separate pre- and post-amputation interventions.

The rehabilitation process involved multiple interventions, and the relative contribution of each modality to the observed improvement could not be determined. Although the patient reported particularly meaningful relief from acupuncture and mirror therapy, these observations reflect his subjective experience and do not establish the independent efficacy of either intervention.

## Figures and Tables

**Table 1:** Physical therapy interventions included in the multidisciplinary rehabilitation program. The rehabilitation protocol comprised low-level laser therapy (LLLT), therapeutic ultrasound, transcutaneous electrical nerve stimulation (TENS), pulsed magnetotherapy, and acupuncture. The figure summarizes the treatment parameters, device settings, and duration of each intervention. All electrotherapy modalities were administered using the BTL-4000 SMART device (BTL Industries, Belgrade, Serbia) according to standardized protocols. Acupuncture was performed on the right hand and left forearm using predefined auricular and body acupoints. Session duration was 5 min for LLLT, ultrasound, and magnetotherapy, 10 min for TENS, and 20 min for acupuncture.

Therapeutic Intervention	Applied Techniques	Duration per Treatment
Low-level laser therapy (LLLT)	Device: BTL-4000 SMART Shape: Continuous Wavelength: 830 nm infrared Frequency: 5000 Hz Energy dose: 4–12 J/cm^2^ Laser class: 3B	5 min
Therapeutic ultrasound	Device: BTL-4000 SMART Shape: Pulsed Frequency: 1 MHz Power: 3.2 W Intensity: 0.2–0.5 W/cm^2^	5 min
Transcutaneous electrical nerve stimulation (TENS)	Device: BTL-4000 SMART Shape: standard Frequency: 100 Hz Pulse width: 100 µs	10 min
Magnetotherapy	Device: BTL-4000 SMART Magnetic field mode: pulse train Magnetic pulse shape: extended rectangular Pulse frequency: 0 to 166 Hz ± 5% Intensity: 54 ^mT^/_10_	5 min
Acupuncture	Acupuncture right hand and left forearm Acupuncture points: ear: Shen Men; thalamus (point 27); PC 7; TE 5; LI 3; SI 11; SI 4; puncta externa 28	20 min

**Table 2:** Long-term Clinical and Rehabilitation Outcomes.

Domain	Outcome	Status
Pain intensity	NRS 3–5 (pre-amputation NRS 7–9)	✓ Improved
Phantom limb pain	Occasional phantom limb pain of mild to moderate intensity	✓ Controlled
Residual limb pain	Stable residual limb pain primarily associated with prosthesis weight and use	✓ Stable
Pharmacological treatment	Gradual tapering of analgesic medication; pregabalin and duloxetine discontinued 2 months after amputation, with progressive reduction of opioid analgesics	✓ Reduced
Functional rehabilitation	Successful postoperative rehabilitation including mirror therapy, residual limb desensitization, scar mobilization, and range-of-motion exercises	✓ Completed
Prosthetic rehabilitation	Ongoing functional adaptation with prosthesis use in daily activities	✓ Improved
Return to work	Return to an adapted workplace compatible with the patient’s functional abilities and disability following prosthetic rehabilitation	✓ Achieved
Psychological rehabilitation	Continued follow-up by psychologist and psychiatrist for PTSD and psychosocial adaptation	↺ Ongoing
Multidisciplinary follow-up	Regular follow-up by the pain specialist with periodic physical therapy and continuous multidisciplinary rehabilitation	↺ Ongoing

## Data Availability

The data supporting the findings of this case report are available from the corresponding author upon reasonable request. No publicly available datasets were generated or analyzed.

## Abbreviations

The following abbreviations are used in this manuscript:

ATP	Adenosine triphosphate
CCEI	Crown–Crisp Experiential Index
CRPS	Complex Regional Pain Syndrome
FDS	Flexor digitorum superficialis
GABA	Gamma-aminobutyric acid
KNT	Nonverbal Cognitive Intelligence Test
LLLT	Low-level laser therapy
MMPI-201	Minnesota Multiphasic Personality Inventory-201
MoCA	Montreal Cognitive Assessment
MT	Mirror therapy
NRS	Numerical Rating Scale
PBM	Photobiomodulation
PEMF	Pulsed electromagnetic field therapy
PLP	Phantom limb pain
PRWE	Patient-Rated Wrist Evaluation
PTSD	Posttraumatic stress disorder
Quick DASH	Quick Disabilities of the Arm, Shoulder and Hand
TENS	Transcutaneous electrical nerve stimulation
TSI	Trauma Symptom Inventory
VAS	Visual Analog Scale
